# The Application of Human Comprehensive Development Theory and Deep Learning in Innovation Education in Higher Education

**DOI:** 10.3389/fpsyg.2020.01605

**Published:** 2020-07-23

**Authors:** Jia-Lin Zhang

**Affiliations:** School of Marxism, Liaoning University, Shenyang, China

**Keywords:** human comprehensive development theory, deep learning, innovation education in higher education, evaluation model, psychology and values

## Abstract

Since the core of talent training in modern society has changed from allowing students to master knowledge to adapting them to a lifelong learning society, this paper studied the innovation education in higher education by combining human comprehensive development theory and deep learning. First, this paper described the training needs of innovative education talents. Second, based on the human comprehensive development theory and deep learning, it puts forward views on innovative quality education in colleges and evaluates the students’ deep learning status by designing a deep learning evaluation model. From the perspective of students, a questionnaire for student-side use was designed according to the deep learning evaluation scale so that students could understand their deep learning status, make improvements based on their current situation, and improve the level of deep learning, thereby obtaining the inspiration of innovation education in higher education. The research shows that the students’ mastery of knowledge and skills is the highest, indicating that the mastery of students in this respect has reached the standard. Although the mean value obtained by the process and method is greater than the median, it is almost the same as the median, which indicates that the students’ mastery of the process and method is still lacking and further efforts are needed. In terms of psychology and values, the mean value is the lowest among the three dimensions and less than the median, indicating that students are the weakest in this respect and further improvement is required. In addition, a new teaching evaluation method was designed based on human comprehensive development theory and deep learning, contributing to innovation education in higher education in China.

## Introduction

With the rapid economic and social development in China in the recent years, people have a new understanding of talent training, and the concept of talent training has also changed to a certain degree (Wang et al., 2015). The core of talent training in modern society has changed from allowing students to master knowledge to adapting them to a lifelong learning society. They need to develop corresponding attitudes to master knowledge and skills, knowledge transformation ability, critical thinking ability, and ability to solve practical problems (Chen J.S., 2018; Fryer and Vermunt, 2018). These coincide with the learning methods advocated by deep learning and human comprehensive development theory. Although the importance of deep learning and comprehensive development has been emphasized in the higher education environment, the application of deep learning in the actual education process is still too weak, and it has not reached the goal of allowing students to develop comprehensively (Cannatella and Cannatella, 2018). Therefore, striving to achieve the goals of deep learning and comprehensive development is one of the important goals of the education community, and teaching evaluation will become an important catalyst in this development path. Teaching evaluation may enable teachers to clearly understand their problems in teaching and actively adjust teaching methods to achieve the purpose of improving teaching quality (Thomas, 2018). Teaching evaluation, as an indispensable part of the teaching process, can be used as a guarantee of teaching foundation and can promote the reform process of deep learning and comprehensive development. At present, although China has already implemented teaching evaluation, the intended purpose cannot be achieved due to great shortcomings in the methods and the modes of teaching evaluation because such methods and modes fail to evaluate the teaching methods of teachers and the deep learning level of students (Carter, 2018).

In the 21st century, innovation has become an inseparable part of people’s lives. With the development of the times and the needs of practice, the state and society attach great importance to the cultivation of innovative and entrepreneurial talents. As an important part of China’s higher education, colleges undertake an important mission in promoting the implementation of innovative and entrepreneurial development strategies, helping to build an innovative country and training high-quality talents for the country. As a training ground for high-quality talents, colleges need to adjust educational policies as soon as possible and coordinate the relationship between innovation and entrepreneurship education with professional education (Wu et al., 2020). Therefore, how to organically integrate innovation and entrepreneurship education with professional education so that they can work together to cultivate students of higher quality as well as fuller employment and entrepreneurship is an urgent problem facing the development of colleges. In recent years, with the rapid development of these innovation and entrepreneurship education, professional education also has a good knowledge accumulation. Policies related to the integration of innovation and entrepreneurship education with professional education issued by the state are more targeted and instructive. The integration of double innovation education and professional education in colleges is an important part of deepening teaching reform based on the needs of talents in the construction of an innovative country. As a result, a new teaching evaluation method was designed in this study based on human comprehensive development theory and deep learning, contributing to innovation education in higher education in China.

## Literature Review

Deep teaching theory plays an important role in guiding teaching in the actual classroom, and it also has a huge role in promoting student learning. However, the investigation is still in its infancy. There are not many theoretical works on it in China, and the understanding in practice is still insufficient. It is necessary to conduct a holistic discussion on it to realize its guidance and promotion in practice. The ultimate goal of deep learning is to achieve the development of higher-order thinking and the formation of real problem-solving capabilities. Therefore, deep learning as an effective way to implement the development of students’ core qualities will inevitably become the trend of curriculum reform and development. In classroom teaching, teachers are the main guides for students’ deep learning, but the current situation of students’ deep learning is not optimistic. How teachers promote the deep learning of students in teaching has become a widely explored subject in the education community.

For deep learning, a large number of worldwide scholars have conducted research. Asikainen and Gijbels (2017) conducted a longitudinal research on the development of learning methods of students in higher education, and the results did not indicate that the students had entered a state of deep learning. Therefore, for deep learning, it is still in the initial implementation stage and requires vigorous development and research. Platow et al. (2013) studied the differences between students’ deep learning and ordinary learning and found that after the students used the deep learning method to study psychology in the first semester, the psychologists’ social identification with students was increased in the second semester. Therefore, the deep learning method will increase students’ social identity to make them more autonomous in learning (Platow et al., 2013). Sulis et al. (2019) evaluated the teaching quality of universities by analyzing the methods set in the framework of generalized mixed models and considered the effects of potential confounding factors outside the process being evaluated, the structure of dependencies between units in the same cluster, the assessment of the actual improvement of the lecturer performance over time, and the use of overall indicators to evaluate the uncertainty related to the overall level of teaching quality (Sulis et al., 2019). Skedsmo and Huber (2018) studied the evaluation methods of teaching quality and found that the student growth percentage model could better evaluate the teaching quality of colleges, yet the evaluation of college teaching quality had a great relationship with the regions, indicating that the evaluation models should vary in different regions. Although there are many investigations on deep learning for students, there are limited investigations in line with China’s national conditions and related to innovation education. For the abovementioned reasons, deep learning and innovation education in higher education are explored.

According to the human comprehensive development theory and deep learning, the views on innovative quality education in colleges were proposed. Also, students’ deep learning status was evaluated through the design of a student deep learning evaluation model. From the perspective of students, according to the deep learning evaluation scale, a questionnaire for student-side use was designed so that students could understand their deep learning status. Then, they could make improvements based on their current situation, improve the level of deep learning, and thus obtain the inspiration of innovation education in higher education.

## Materials and Methods

### Requirements for Talent Training in Innovation Education

Innovation education is a new type of talent training. At present, China attaches great importance to innovation education, and how to carry out innovation education has become the direction of the efforts of personnel in the education industry (Clifford and Montgomery, 2015). Based on the research content and research results of various scholars, this paper summarizes the capabilities of talents required for innovation education in the following aspects:

(1) Innovation and creativity: The ability of innovation and creativity is one of the essential conditions for talents to carry out innovation activities. It is a concentrated expression of excellent qualities such as developed intelligence, rich experience, and perfect personality. It is also a kind of ability with strong divergent thinking. Based on the original experience and ability, it is extended from the existing thinking to generate new ideas or thinking. This process is from quantitative change to qualitative change. The creation of innovative and creative abilities requires college students to have psychological construction prerequisites such as strong curiosity, strong willpower, and aggressiveness. Then, students need to have abundant knowledge reserves. Only on the basis of rich knowledge can students’ thinking change from quantitative to qualitative, and students’ intelligence must be higher.
(2) Criticism: Critical ability is the thought or ability to reflect on or even question what people have learned. To gain critical ability, students must have the ability to explain and analyze everything and make inferences. Students also need to have an understanding of things and to analyze new things based on this, thereby making critical decisions. Students should also have the ability to self-calibrate and conduct self-reflection. In the face of their wrong thinking, timely proofreading and correction are needed to form a virtuous circle.
(3) Independence: With the rapid development of the Internet, students are in the ocean of information every day. A large amount of positive or negative information, whether correct or wrong, degrades students’ ability to think independently. The ability to think independently is the source of all innovative activities. Without it, the country will face a sharp decline in innovative talents. With the rapid rise of artificial intelligence, one’s ability to think independently has become one of the most important factors that distinguish it from artificial intelligence.
(4) Practice: Practice is based on sound thinking. The ideas are put into practice by using knowledge, intelligence, and acquired skills. The development of practical ability requires students to have the ability to perceive things and to think directly or indirectly about knowledge. It also requires students to have the ability to integrate knowledge. It means that they can integrate what they have learned to form a specific knowledge system, thereby using knowledge to achieve the goal of practice.

### Human Comprehensive Development Theory and Innovation Education

The human comprehensive development theory is an important guideline and educational philosophy in China’s college education. It is the premise and the theoretical basis of innovative quality education for college students. It is also the highest ideal pursued by the education community, so the education of college students should be based on the human comprehensive development theory. The human comprehensive development theory is a manifestation of human nature. All education that suppresses and deprives human nature is contrary to social laws and it is incorrect (Wang and Wang, 2018). The human comprehensive development is the premise and theoretical basis of innovative quality education for college students. At present, the innovative quality education for college students is still in the training stage. Although China’s education community has recognized the importance of innovative quality education, it is not ideal for its implementation and effect (Yuan and Cai, 2018).

The comprehensive development of human personality includes the overall improvement of individual subjectivity and the enrichment of individual uniqueness. The improvement of individual subjectivity requires people to give full play to their subjective initiative and creativity as well as actively participate in practical activities and social relations, promoting social development and the overall development of individuals. The increase and the enrichment of individual uniqueness require individuals to maintain and pursue independent personality and ideals. No one is the same. It is the increase and enrichment of individual uniqueness that have produced the present colorful world.

Currently, China’s teaching concept of innovative quality education for college students has undergone a great change. Colleges and families have attached great importance to the innovation consciousness and ability development of students and have begun to affect students to a certain extent. To carry out innovative quality education for students and train their innovative quality, major colleges begin to implement new teaching models and set up various encouragement funds. However, China’s education concept is deeply entrenched, and it is difficult to change it in a short time. As a consequence, the current innovative quality education method for college students is still relatively old-fashioned, and the teaching materials are relatively backward. Also, the teachers have neglected the students’ initiative and focused more on the “teaching” process. In addition, college’s understanding of innovative quality education is not consistent with that of the students. At present, college’s efforts in innovative quality education only stay on activities such as innovative competitions. Nevertheless, the competition cannot be used for innovative quality education, causing students to be afraid of innovation, dare not to innovate, and think that their ability is insufficient. Most of college’s training objects for innovative quality education are “top students” carefully selected by the teacher, which can be reflected in the usual participation in innovative competitions. Thus, the training is seriously imbalanced.

The college should regard students as the subject of innovation. Its role is only to guide, not to limit students’ innovative ideas. The process of students’ innovation is to integrate and learn from what they have learned. Colleges should give strong support to students to make some small inventions. In fact, most colleges do not support such innovation. They are worried that students’ energy will be attracted by these things, and they will not be able to efficiently receive the general education provided by teachers. Moreover, they are afraid that students will harm the majesty of education to some extent. However, this approach will not maintain the majesty of education but will suppress the students’ thoughts and destroy the students’ innovative consciousness (Li, 2017; Zhang and Chen, 2019).

### Deep Learning and Innovation Education

**FIGURE 1:**
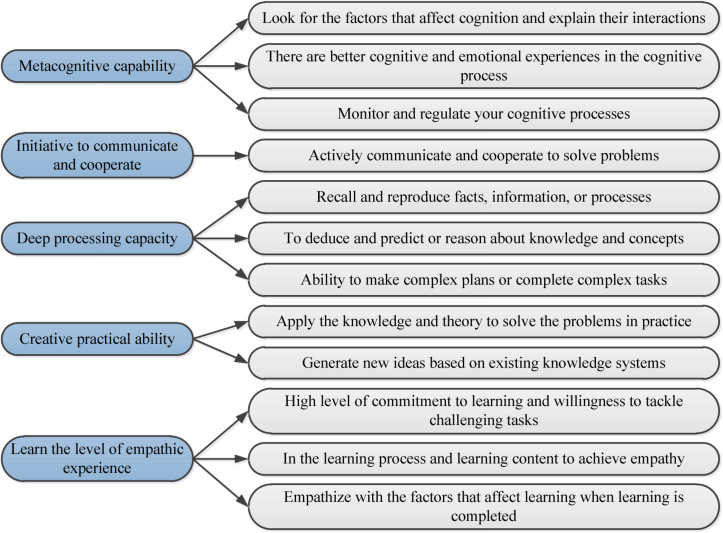
Deep learning dimension diagram.

Deep learning is a learning method that integrates cognition, construction, and migration. It means that scholars have a certain purpose to learn. After learning, they have a comprehensive and in-depth understanding of knowledge and can establish what they have learned into their cognitive structure, thereby forming creative and critical ways of thinking (Jin, 2019). Deep learning can be divided into five dimensions, and the specific connotation of these five dimensions is shown in Figure 1.

Metacognitive capability shows the self-cognition degree of students, in other words, whether students have their plans for learning, whether students can independently complete a learning task and time planning, and whether students can transfer and use knowledge after learning (Dong et al., 2019).

The ability to the initiative to communicate and cooperate shows the situation in which students communicate and cooperate with others in classroom activities, after-class activities, and extra-curricular activities to solve problems.

The deep processing capacity is the concentrated expression of students’ memory, logic, thinking, reasoning, induction, deduction, and understanding ability to show the depth and the breadth of students’ understanding of knowledge.

Creative practical ability is a concentrated expression of students’ creative thinking and practical ability. Only by combining creative thinking and practical ability can the best state of creative performance be achieved.

Learning the level of empathic experience is the embodiment of students’ integration of learning ability and learning emotions. They can think independently in various learning processes to discover their ideals and interests, thereby achieving a learning state with a sense of accomplishment (Sun, 2019).

In the process of innovation education, the learning efficiency of deep learning is much higher than those of other learning methods. Therefore, the innovation education in colleges was discussed through the investigation of deep learning of college students.

### Deep Learning Evaluation Model

On the one hand, the fundamental problem to be solved in the teaching design of deep learning is to work around teaching objectives, teaching methods and strategies as well as teaching activities. On the other hand, the evaluation method is selected. In the evaluation of deep learning, the science must be unified with the objective. Also, student personalization and development are taken as the direction. Moreover, the role of operability and independent overall principles must not be ignored.

**FIGURE 2:**
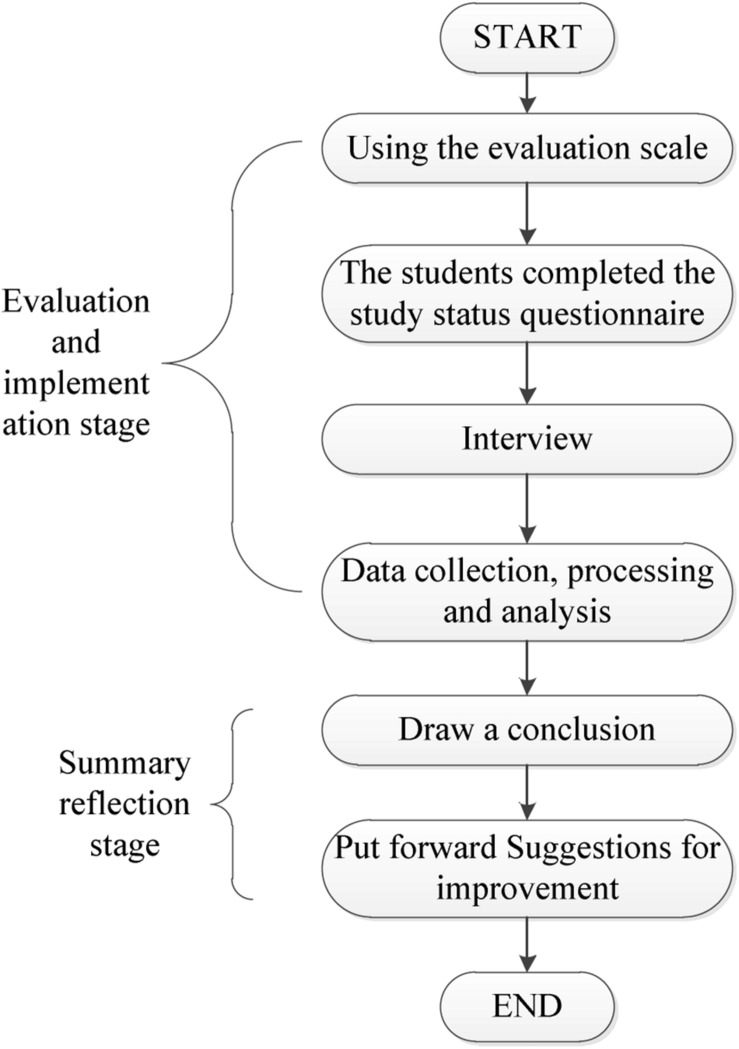
Deep learning evaluation model.

The specific working form of the deep learning model designed in this paper is shown in Figure 2.

First, the students’ deep learning level was evaluated according to the evaluation scale, and the data used were derived from a deep learning survey statistics form filled out by the students. Then, some students were randomly selected for interviews to understand the true ideas that the students did not describe in the deep learning statistics form and to ensure the authenticity of the form. Finally, the obtained data were sorted, evaluated, and analyzed to reach a conclusion (Rogoza et al., 2018). The deep learning evaluation scale is shown in Table 1.

**TABLE 1:** Evaluation scale for deep learning.

Evaluation criterion	Agree to degree				
	Totally disagree	Disagree	General	Agree	In full agreement
(1) No knowledge of problem solving	1	2	3	4	5
(2) Inability to understand the problem properly	1	2	3	4	5
(3) Easily misled by irrelevant knowledge	1	2	3	4	5
(4) Answer questions that are logically confusing	1	2	3	4	5
(5) Answer the question with simple tautology	1	2	3	4	5
(6) Understand problems superficially	1	2	3	4	5
(7) Link a single event to a single clue to a conclusion	1	2	3	4	5
(8) The question disagrees with the conclusion	1	2	3	4	5
(9) Multiple correct information can be obtained from the problem	1	2	3	4	5
(10) Clues can be integrated, but not a complete network	1	2	3	4	5
(11) The same question leads to different conclusions	1	2	3	4	5
(12) Grasp the deep meaning of the problem	1	2	3	4	5
(13) Understand the problem as a whole	1	2	3	4	5
(14) Integrate the clues in the problem into an organic whole	1	2	3	4	5
(15) The conclusion remains consistent in the present situation	1	2	3	4	5
(16) Can analyze the problem deeply and summarize the unknown situation	1	2	3	4	5
(17) Continue to research problems and develop innovative ideas	1	2	3	4	5
(18) The conclusion is open	1	2	3	4	5

### Data Collection

From the perspective of students, a questionnaire for student-side use was designed according to the deep learning evaluation scale so that students could understand their deep learning status, make improvements based on their current situation, and thus improve the level of deep learning.

A total of 350 questionnaires were distributed in this study, 350 questionnaires were recovered, and 31 invalid questionnaires were eliminated. Finally, 319 valid questionnaires were obtained. The data in the valid questionnaires were analyzed and processed to obtain the situation of students’ deep learning. The data obtained from the questionnaire were emailed to 10 relevant experts who studied the deep learning of the students, and the experts were asked to score the various research data to ensure the accuracy of the data. Finally, the experts gave scores based on years of experience and relevant public research questionnaires.

## Results

### Descriptive Analysis of the Data Obtained

**FIGURE 3:**
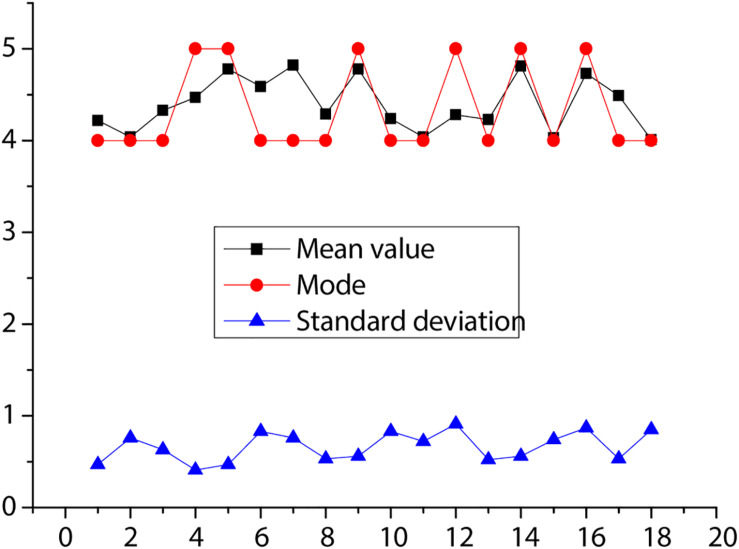
Expert scoring results.

For the deep learning evaluation method of this paper, the expert scoring situation is shown in Figure 3. As shown in the figure, the standard deviation of each item is below 1, which indicates that the deep learning evaluation method is feasible and the opinions of experts are almost unified. The mean score of all indicators is above 4, which indicates that the experts agree with the evaluation system of this paper. It shows that the evaluation method has a certain degree of accuracy.

**FIGURE 4:**
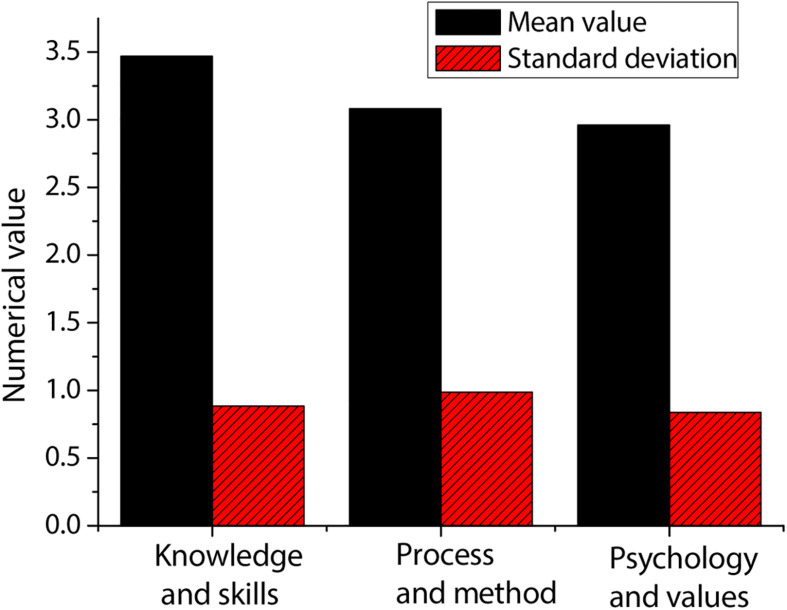
Statistics of students’ deep learning from three dimensions.

The statistics on students’ deep learning are made from three dimensions, namely, knowledge and skills, process and method, and psychology and values. The statistical results are shown in Figure 4. According to the figure, the students’ mastery of knowledge and skills is the highest. The mean value obtained by knowledge and skills is greater than the median of 3, indicating that the students’ mastery of knowledge and skills has reached the standard. Although the mean value obtained by the process and method is greater than the median of 3, it is almost the same as the median of 3, which indicates that the students’ mastery of the process and method is still lacking, and further efforts are needed. In terms of psychology and values, the mean value is the lowest among the three dimensions, and it is less than the median of 3, indicating that students are the weakest in this respect and need further improvement.

Generally speaking, the answers of the students in these three aspects are mostly in the middle or lower position, and they all remain in an uncertain state of description. Therefore, it can be explained in such a way that students’ self-confidence in these three aspects is low or that students have different degrees of deficiencies in these three aspects, and they need to be improved.

**FIGURE 5:**
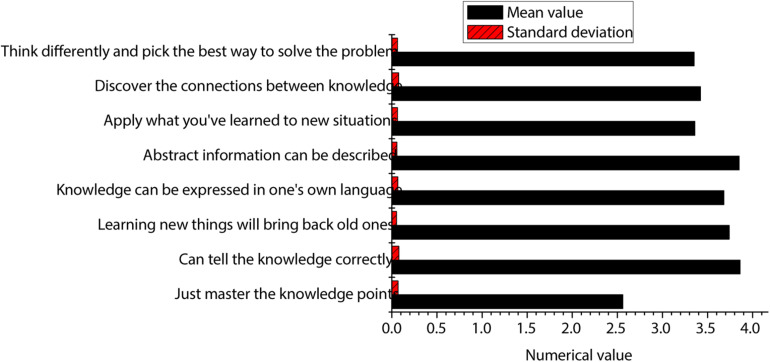
Detailed statistics of knowledge and skills.

The detailed statistics on knowledge and skills are shown in Figure 5. According to the figure, the students’ mastery of knowledge and skills is almost up to standard. In the item of “just master the knowledge points,” the students have the lowest degree of consent, indicating that they have a certain understanding of deep learning. They believe that deep learning is not just about mastering the knowledge points but also to apply the learned knowledge flexibly and integrate them, ultimately supporting students’ ideas for innovation. In the figure, the item of “abstract information can be described” has a high mean value, indicating that students are not too restrictive about the form of knowledge, and they can conduct appropriate autonomous learning. Most students can discover the connection between knowledge, which shows that they have a high ability to construct knowledge and it can provide support for subsequent innovative ideas.

**FIGURE 6:**
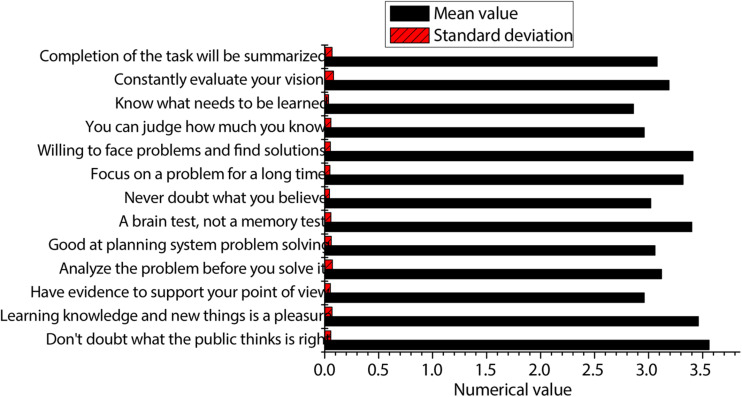
Detailed statistics of the process and method.

The detailed statistics on process and method are shown in Figure 6. According to the figure, the item of “don’t doubt what the public thinks is right” has a higher mean value, greater than the median of 3 and more than the median. The item of “you can judge how much you know” has a lower mean value. It can reflect that the critical ability of students is generally not high. For the things that everyone thinks are correct, they also adopt the attitude of others, without enough ability to think. Thus, students need to be strengthened in this aspect. The majority of people like to be exposed to new things, new knowledge, and the brain test rather than memory test, which indicates that students have a higher acceptance of new knowledge and new things as well as higher brain-thinking ability. The mean score of students who are good at systematic planning and solving complex problems is low, indicating that they have low problem-solving ability and lack systematic thinking. Therefore, the students’ ability to solve problems needs to be improved. The mean score of the students in terms of knowing what needs to be learned is low, which indicates that their active learning ability is relatively lacking, and it is urgent to improve in this aspect. Relying too much on teachers, classmates, or parents will greatly reduce students’ preference for innovation and will obliterate their innovative ideas.

**FIGURE 7:**
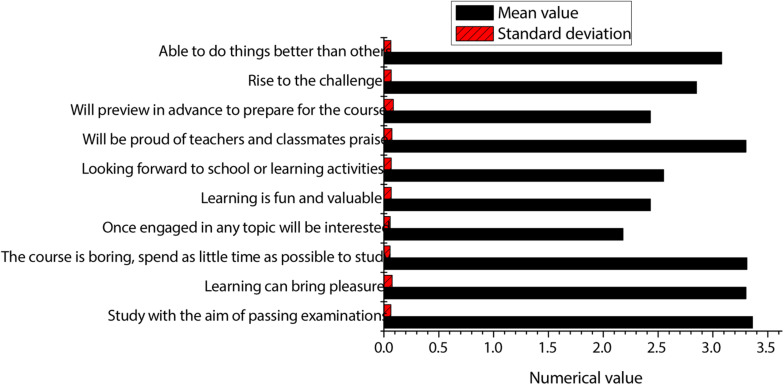
Detailed statistics of psychology and values.

The detailed statistics on psychology and values are shown in Figure 7. According to the figure, the students’ scores are generally lower in terms of psychology and values. From the “will be proud of teachers and classmates praise,” it can be seen that students like a sense of accomplishment to themselves brought about by learning. The sense of accomplishment will also encourage students to study harder. The lower mean score for looking forward to learning activities indicates that students do not like daily learning activities. Moreover, the mean score of the students in the item of “learning is fun and valuable” is low, which shows that they do not have a high degree of recognition for learning, indicating that they do not know why they need to learn. In addition, it can also reflect that college’s education of students does not start from the ideas of students. The higher score of students in the item of “able to do things better than others” indicates that they are more confident in themselves and that they will be willing to study harder when learning brings joy to themselves. However, students have higher mean scores in the “study with the aim of passing examinations” and “the course is boring, spend as little time as possible to study,” which indicate that they still have traditional test-taking concepts for learning and are not studying for the improvement of their ability.

### Correlation Analysis Between Various Dimensions of Deep Learning and Students’ Ability

**FIGURE 8:**
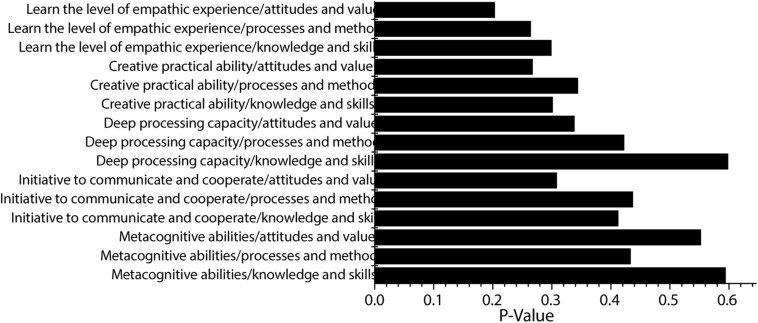
Correlation between students’ ability and deep learning.

The correlation analysis results of various dimensions of deep learning and students’ ability are shown in Figure 8. The figure shows that the correlation coefficient between academic investment level and metacognitive ability is 0.611 (*P* < 0.05), and the correlation between the two is moderately positive. The correlation coefficient between academic investment level and deep processing capacity is 0.601 (*P* < 0.05), and the correlation between the two is moderately positive. The correlation coefficient between academic investment level and teaching process experience is 0.297 (*P* < 0.05), and the correlation between the two is low positive. The correlation coefficient between academic investment level and learning process experience is 0.441 (*P* < 0.05), and the correlation between the two is moderately positive. The correlation coefficient between metacognitive ability and deep processing capacity is 0.520 (*P* < 0.05), and the correlation between the two is moderately positive. The correlation coefficient between metacognitive ability and teaching process experience is 0.341 (*P* < 0.05), and the correlation between the two is low positive. The correlation coefficient between metacognitive ability and learning process experience is 0.451 (*P* < 0.05), and the correlation between the two is moderately positive. The correlation coefficient between deep processing capacity and the teaching process experience is 0.370 (*P* < 0.05), and the correlation between the two is low positive. The correlation coefficient between deep processing capacity and learning process experience is 0.401 (*P* < 0.05), and the correlation between the two is low positive. The correlation coefficient between the teaching process experience and the learning process experience is 0.302 (*P* < 0.05), and the correlation between the two is low positive.

If students want to get the highest level of deep learning, which is the level of empathic experience, they need to improve in the three dimensions of knowledge and skills, processes and methods, and attitudes and values.

### Case Analysis of Students

In this paper, three students who participated in the questionnaire survey, with the grade in the top 5, 20–50, and 50–80%, were selected for the interview. The interview results are shown below.

Through interviews with students, it was found that the student with better grades is more capable of active learning. He/she actively links old knowledge with new knowledge to form his/her knowledge system and cognitive structure. The student can also almost use the knowledge to solve problems encountered in real life, and he/she has almost reached the level of deep learning. The student with a moderate grade prefers to study and can also connect new knowledge with old knowledge. He/she will preview before class and continue to study the content of interest after class, but the student is greatly affected by his/her interests. In the part that he/she is interested the student can almost reach the level of deep learning, while in the part that he/she is not interested the student will show a more passive state. He/she also lacks the ability to criticize. He/she does not question the accuracy of what everyone thinks is right. Although the student whose grade was in the middle and lower level can study hard in the classroom, he/she will not take the initiative to explore when he/she encounters learning difficulties. During the study, he/she rarely participates in discussion activities and does not reflect on his/her mistakes.

From the above research, it can be found that students with good grades will actively explore, discover, and study the knowledge they are interested in. However, the students with lower grades are limited to what the teacher says and what the textbooks describe. They rarely learn autonomously. Most of them are also in the state of mechanical learning in the traditional test-oriented education.

## Discussion

The core of talent training in modern society has changed from allowing students to master knowledge to adapting them to a lifelong learning society. They need to have corresponding attitudes to master knowledge and skills, knowledge transformation ability, critical thinking ability, and ability to solve practical problems. Thus, this paper studies and analyzes the deep learning situation of college students (Elliott, 2018).

The research shows that the students’ mastery of knowledge and skills is the highest, and the mean value obtained by knowledge and skills is greater than the median, indicating that the mastery of students in this respect has reached the standard. Although the mean value obtained by the process and method is greater than the median, it is almost the same as the median, which indicates that the students’ mastery of the process and method is still lacking, and further efforts are needed. In terms of psychology and values, the mean value is the lowest among the three dimensions, and it is less than the median, indicating that students are the weakest in this respect and need further improvement (Shen and Ho, 2020). Generally speaking, the answers of the students in these three aspects are mostly in the middle or lower position, and they all remain in an uncertain state of description. Therefore, it can be explained in such a way that the students’ self-confidence in these three aspects is low or that students have different degrees of deficiencies in these three aspects, and they need to be improved (Qian et al., 2018). Students with good grades will actively explore, discover, and study the knowledge they are interested in. However, the students with lower grades are limited to what the teacher says and what the textbooks describe. They rarely learn autonomously. Most of them are also in the state of mechanical learning in the traditional test-oriented education (Chen, 2019).

The critical ability of students is generally not high. For the things that everyone thinks are correct, they also adopt the attitude of others, without enough ability to think. Critical thinking is the most important thinking in deep learning, which is almost consistent with the research results of deep learning for students by scholars Wang et al. (2015).

In this paper, the research results show that college students are not capable of processing information and can only do simple processing or not processing what they learn daily. There are few students who can achieve a thorough understanding of what they have learned, indicating that the deep learning situation of college students is poor, and only a few students can reach the status of deep learning (Shen et al., 2019). Nevertheless, it is not entirely the students’ reason. First, college’s teaching methods do not meet the standards of deep learning. College does not guide students to deep learning but “instill” traditional knowledge. Therefore, the students’ learning status is mostly passive acceptance. Students lack the consciousness of active learning, and college does not emphasize the training of students’ active consciousness. In addition, college’s learning atmosphere is not strong. Thus, colleges should make corresponding efforts to improve the learning atmosphere and adjust teaching methods (Wu and Song, 2019).

Teachers in colleges should play a role in driving students. The teacher’s investment in students is positively related to the students’ learning status, but at present, college teachers’ investment in students is not enough due to the pressure of scientific research. It leads to indifferent teacher–student relationships in colleges. The teachers do not have a strong influence on students and do not play a guiding role (Chen M, 2018; Wu et al., 2019). In addition to school relationships, students should also change their perceptions of learning. Learning is not to cope with the exam but to acquire the ability to solve problems and the ability to think about problems. Therefore, students should pay attention to learning to achieve more results with less effort (Zheng et al., 2015; Zheng and Liu, 2020).

## Conclusion

In this paper, innovation education in higher education was studied by combining the human comprehensive development theory with deep learning. The study shows that the education methods of colleges cannot achieve the purpose of deep learning for students, and the learning status of students is almost the same as that of exam-oriented education. College students are not capable of processing information and can only do simple processing or not processing what they learn daily. There are few students who can achieve a thorough understanding of what they have learned, indicating that the deep learning situation of college students is poor, and only a few students can reach the status of deep learning. Although some achievements have been made in this research, there are still some shortcomings. The research data included in this paper are few and not comprehensive. For deep learning, it has a strong relationship with the college situation, but this paper only studies a small number of students in a college. The results are not applicable to every college or every region. Therefore, the next step is to carry out studies in other colleges and other regions based on the research method of this paper to provide more data support for the research results of this paper.

## Data Availability Statement

All datasets generated for this study are included in the article/supplementary material.

## Ethics Statement

The studies involving human participants were reviewed and approved by Liaoning University Ethics Committee. The patients/participants provided their written informed consent to participate in this study.

## Author Contributions

The author confirms being the sole contributor of this work and has approved it for publication.

## Conflict of Interest

The author declares that the research was conducted in the absence of any commercial or financial relationships that could be construed as a potential conflict of interest.
